# Primary motor cortex of the parkinsonian monkey: altered neuronal responses to muscle stretch

**DOI:** 10.3389/fnsys.2013.00098

**Published:** 2013-11-26

**Authors:** Benjamin Pasquereau, Robert S. Turner

**Affiliations:** Department of Neurobiology, Center for Neuroscience and The Center for the Neural Basis of Cognition, University of PittsburghPittsburgh, PA, USA

**Keywords:** stretch reflex, primary motor cortex, MPTP, Parkinson's disease, rigidity

## Abstract

Exaggeration of the long-latency stretch reflex (LLSR) is a characteristic neurophysiologic feature of Parkinson's disease (PD) that contributes to parkinsonian rigidity. To explore one frequently-hypothesized mechanism, we studied the effects of fast muscle stretches on neuronal activity in the macaque primary motor cortex (M1) before and after the induction of parkinsonism by unilateral administration of 1-methyl-4-phenyl-1,2,3,6-tetrahydropyridine (MPTP). We compared results from the general population of M1 neurons and two antidromically-identified subpopulations: distant-projecting pyramidal-tract type neurons (PTNs) and intra-telecenphalic-type corticostriatal neurons (CSNs). Rapid rotations of elbow or wrist joints evoked short-latency responses in 62% of arm-related M1 neurons. As in PD, the late electromyographic responses that constitute the LLSR were enhanced following MPTP. This was accompanied by a shortening of M1 neuronal response latencies and a degradation of directional selectivity, but surprisingly, no increase in single unit response magnitudes. The results suggest that parkinsonism alters the timing and specificity of M1 responses to muscle stretch. Observation of an exaggerated LLSR with no change in the magnitude of proprioceptive responses in M1 is consistent with the idea that the increase in LLSR gain that contributes to parkinsonian rigidity is localized to the spinal cord.

## Introduction

Rigidity, one of the cardinal signs of Parkinson's disease (PD), is defined clinically as a sustained increase in resistance to passive movement of a joint throughout its range (Stebbins and Goetz, 1998). Although various possible contributing factors have been studied for decades at the central (Cantello et al., 1991; Aminoff et al., 1997; Strafella et al., 1997; Young et al., 1997), spinal (Angel and Hofmann, 1963; Dietrichson, 1971; Andersson and Sjolund, 1978; Delwaide et al., 1991; Lelli et al., 1991; Marchand-Pauvert et al., 2011) and muscle (Dietz et al., 1981; Lee and Tatton, 1982; Noth et al., 1988) levels, the exact pathophysiologic mechanisms of parkinsonian rigidity remain elusive. Many observations have suggested that the stretch reflex plays a key role in the generation of rigidity, but controversies persist regarding the precise relationship (Lee and Tatton, 1975; Berardelli et al., 1983; Rothwell et al., 1983; Meara and Cody, 1992; Xia et al., 2009).

In neurologically-intact animals, the stretch reflex appears to regulate limb stiffness to maintain precise control of multi-joint posture during interactions with an unstable environment (Rothwell, 1990; Shemmell et al., 2010; Pruszynski et al., 2011a). The stretch reflex involves at least two components: a short-latency response mediated by fast-conducting segmental pathways (Magladery et al., 1951; Burke et al., 1984), and a long-latency component mediated, at least in part, by a transcortical pathway (Hammond, 1955; Marsden et al., 1972; Capaday et al., 1991; Day et al., 1991; Matthews, 1991; Palmer and Ashby, 1992; Pruszynski et al., 2011b). The idea that cortex contributes to the long-latency component (i.e., the long latency stretch reflex, LLSR) is supported by observations that neurons in primary motor cortex (M1) respond to proprioceptive perturbations at delays (20–60 ms prior to the late muscle reaction) appropriate for them to participate in this long-latency component (Evarts, 1973; Conrad et al., 1975; Evarts and Tanji, 1976; Cheney and Fetz, 1984; Abbruzzese et al., 1985; Aminoff et al., 1997; Mackinnon et al., 2000). The ability of unilateral muscle stretches to evoke bilateral LLSRs in cases of congenital corticospinal tract malformation (Matthews et al., 1990; Capaday et al., 1991) provides strong support for the view that a component of the LLSR is mediated via a transcortical route. It is important to note, however, that slow-conducting spinal reflex pathways also contribute to the LLSR (Berardelli et al., 1983; Cody et al., 1986).

In PD, the short-latency component of the stretch reflex appears essentially normal (Berardelli et al., 1983; Rothwell et al., 1983; Cody et al., 1986; Meara and Cody, 1993; Hayashi et al., 2001), but the LLSR is markedly exaggerated (Tatton and Lee, 1975; Mortimer and Webster, 1979; Berardelli et al., 1983; Rothwell et al., 1983; Cody et al., 1986; Meara and Cody, 1993; Hayashi et al., 2001; Xia et al., 2009). By increasing the activation of muscles that oppose passive stretch, an abnormally-increased LLSR may be a key factor in the genesis of parkinsonian rigidity. Indeed, several studies have documented a correlation between the magnitude of the LLSR and the severity of parkinsonian rigidity (Lee and Tatton, 1975; Mortimer and Webster, 1979; Tatton et al., 1984; Xia et al., 2009). Other studies failed to confirm such a clear relationship (Berardelli et al., 1983; Rothwell et al., 1983; Cody et al., 1986), most likely because additional reflex abnormalities, such as the aberrant activation of the shortening muscle, also contribute (Andrews et al., 1972; Xia et al., 2006, 2009). Further support for the LLSR's role in parkinsonian rigidity comes from observations that treatments for PD (i.e., dopaminergic medication, pallidotomy and subthalamic stimulation) clearly reduce stretch-related muscled activation, and ameliorate muscle stiffness proportionately (Teravainen et al., 1989; Limousin et al., 1999; Fung et al., 2000; Hayashi et al., 2001; Lee et al., 2002; Xia et al., 2006; Levin et al., 2009; Marchand-Pauvert et al., 2011; Raoul et al., 2012).

Two straightforward possibilities may account for the exaggerated LLSR of PD. The trans-cortical hypothesis states that abnormal neuronal activity transmitted from the parkinsonian basal ganglia (BG) (Delong and Wichmann, 2007) causes increased somatosensory responsiveness in the M1 neurons that participate in the LLSR. Indeed, neurons in the BG and thalamus of parkinsonian subjects have larger-than-normal responses to proprioceptive stimulation and reduced response selectivity with respect to the joint and limb stimulated (Filion et al., 1988; Bergman et al., 1994; Pessiglione et al., 2005). According to this model, increased proprioceptive responsiveness might be transmitted to M1 where it would appear as (1) a greater incidence of neuronal responses to proprioceptive stimulation in the population of M1 neurons, (2) an increase in the balance of torque-evoked increases over decreases, or (3) an increase in the magnitude of torque-evoked responses in individual M1 neurons. It is unclear, however, how this simple model can be reconciled with the abundant evidence that the M1 of parkinsonian subjects has reduced responsiveness to somatosensory inputs as measured by electroencephalography (Rossini et al., 1989; Aminoff et al., 1997; Rickards and Cody, 1997; Lewis and Byblow, 2002; Schrader et al., 2008; Degardin et al., 2009), transcranial magnetic stimulation (Lewis and Byblow, 2002), and functional imaging (Boecker et al., 1999). The alternative model hypothesizes that the exaggerated LLSR of PD is mediated by abnormal function of slow-conducting spinal reflex pathways (Berardelli et al., 1983; Cody et al., 1986; Simonetta Moreau et al., 2002; Marchand-Pauvert et al., 2011; Raoul et al., 2012).

Primary motor cortex is composed of a complex collection of distinct cell types that differ with respect to intrinsic physiology (McCormick et al., 1985; Connors and Gutnick, 1990; Stewart and Foehring, 2000; Hattox and Nelson, 2007; Kiritani et al., 2012) and afferent innervation (Swadlow, 1994). These subtypes may perform dissimilar functions in the LLSR and may be affected differently in the parkinsonian state (Pasquereau and Turner, 2011). For example, distant-projecting lamina 5b pyramidal tract-type neurons (PTNs) and intratelencephalic-projecting corticostriatal neurons (CSNs) in the M1 have markedly different somatosensory responsiveness (Bauswein et al., 1989; Turner and Delong, 2000). PTNs are positioned to play a relatively direct role in the expression of the abnormal LLSR in PD due to their direct projections to segmental motor nuclei (Landgren et al., 1962; Brodal, 1978; Kuypers, 1981). The CSNs of M1, in contrast, provide an important glutamatergic input to motor regions of the striatum, and thus, are in a position to influence, for better or worse, the disordered physiology of the dopamine-depleted striatum (Mallet et al., 2006).

Despite clear implication of the motor cortex in the LLSR, only a few studies have compared cerebral responses to proprioceptive inputs and abnormalities in the LLSR in parkinsonian subjects (Rossini et al., 1989, 1991; Aminoff et al., 1997), and these used a non-invasive electrocerebral approach. These studies reported the seemingly paradoxical finding that an exaggerated LLSR was correlated with *attenuation* of a sensory evoked potential that is thought to emanate from precentral cortical areas. To elucidate the changes in cortical function associated with the exaggerated LLSR of PD, we performed single unit recording in the M1 of parkinsonian macaques and studied the short latency neuronal responses to rapid muscle stretch. Based on previous observations that the spontaneous activity of PTN and CSN populations are affected differently in parkinsonism (Mallet et al., 2006; Pasquereau and Turner, 2011), we hypothesized that the responses of PTNs and CSNs to muscle stretch would be affected differently in parkinsonism. To address this issue, antidromically-identified neurons (PTNs and CSNs) were studied in the arm area of M1 in two rhesus monkeys. The stretch reflex was evoked by sudden rotations of the animal's elbow or wrist and recordings were obtained before and after induction of parkinsonism by unilateral intra-carotid administration of 1-methyl-4-phenyl-1,2,3,6-tetrahydropyridine (MPTP).

## Methods

### Animals, apparatus, tasks

Two female monkeys (*Macaca mulatta*) were used for these experiments (monkeys V and L). All aspects of animal care were in accord with the *Guide for the Care and Use of Laboratory Animals* (National Research Council, 1996), and all procedures were approved by the institutional animal care and use committee.

Data from these animals were part of a recent publication describing changes in resting cortical activity associated with parkinsonism (Pasquereau and Turner, 2011). Many aspects of the experimental approach were described in detail in that report. In brief, the animals performed a visuomotor step-tracking task similar to one used in previous studies of cortical and BG neuronal activity (Alexander, 1987; Mitchell et al., 1987; Alexander and Crutcher, 1990; Turner and Delong, 2000). The animal sat in a primate chair and faced a computer monitor. The right arm was secured into a close-fitting padded cradle attached to a one-dimensional torquable manipulandum. The wrist (monkey L) or elbow (monkey V) joint was aligned with the manipulandum's axis of rotation. Flexion and extension movements rotated the manipulandum in the horizontal plane and thereby controlled the horizontal position of an onscreen cursor. A trial began when a center target appeared and the monkey made the appropriate joint movement to align the cursor with the target. The monkey maintained this position for the duration of a start-position hold period (random duration, 2–5 s), during which the animal could not predict the location of the upcoming lateral target. The target then shifted to the left or right (chosen at random), and the animal moved the cursor to capture the lateral target. The animal received a drop of juice for successful completion of the task.

On two-thirds of the trials (selected at random), single flexing or extending torque impulses (0.1 Nm–50 ms duration) were applied to the manipulandum by a DC brushless torque motor (TQ40W, Aerotech Inc., Pittsburgh PA) at an unpredictable time beginning 1–2 s (uniform randomized distribution) after initial capture of the center target. Each square-wave torque impulse induced an angular displacement of the joint (mean = 10-deg) causing a sudden stretch of arm extensor or flexor muscles. The animals were not trained to produce a specific response to these unpredictable perturbations, but the animals naturally adopted a strategy that returned the joint to its initial pre-impulse position.

Later aspects of the behavioral trial, which evaluated instruction-related neuronal activity, are irrelevant to the current study.

### Surgery

The animals were prepared surgically using aseptic techniques under Isoflurane inhalation anesthesia (Pasquereau and Turner, 2011). A cylindrical stainless steel chamber was implanted at an angle of 35° in the coronal plane to allow access to the arm-related regions of the left M1 and the posterior putamen. The chamber and hardware for head fixation were fixed to the skull with bone screws and methyl methacrylate polymer.

Pairs of fine Teflon-insulated multistranded stainless steel wires were implanted into multiple arm muscles: flexor carpi ulnaris, flexor carpi radialis, biceps longus, brachioradialis, and triceps lateralis in monkey L; and posterior deltoid, trapezius, triceps longus, triceps lateralis and brachioradialis in monkey V. The wires were led subcutaneously to a connector fixed to the skull implant. Accurate placement of electromyographic (EMG) electrodes was verified post-surgically. Following surgery, animals were given prophylactic antibiotics and analgesic medication.

### Placement of electrodes for antidromic identification

PTNs and CSNs were identified by antidromic activation from electrodes implanted in the cerebral peduncles and posterolateral striatum. Sites for implantation were identified using standard electrophysiological mapping techniques (Turner and Delong, 2000). Arm-related areas of the putamen were identified by sensorimotor examination of striatal activity and microstimulation effects. The arm-related fiber tract in the pre-pontine cerebral peduncle (ventral to the substantia nigra) was located using similar techniques.

Custom-built PtIr microwire electrodes were implanted at arm-related sites in putamen and the peduncle (Turner and Delong, 2000). After implantation, stimulation through the electrodes evoked arm movements similar to those observed at the target sites during microelectrode mapping. In both animals, three such electrodes were implanted in the posterior putamen between the planes of HC anterior 8 and 14, and one electrode was implanted in the arm-responsive portion of the pre-pontine peduncle (for details, see Turner and Delong, 2000). Histologic reconstruction confirmed that the striatal and peduncle electrodes were at sites known from anatomical studies to receive the bulk of M1 CSN and PTN projections, respectively (Brodal, 1978; Flaherty and Graybiel, 1991; Takada et al., 1998; Turner and Delong, 2000).

### Data acquisition

Areas of M1 related to the primary joint used in the task were identified using microstimulation and sensorimotor mapping. We preformed trans-dural extracellular recording using single glass-coated PtIr microelectrodes mounted in a hydraulic microdrive (MO-95, Narishige Intl., Tokyo). A cortical region was targeted for data collection if neurons responded to active and/or passive movement of the arm and microstimulation at low currents evoked contraction of forelimb muscles (<40-μA, 10 biphasic pulses at 300-Hz). Microelectrode penetrations were performed throughout the targeted cortical area using sequential stimulation of each putamen and peduncle stimulating site as search stimuli (biphasic current pulses of 700-μA, 0.2-ms duration separated by 0.1-ms, >1.5-s between successive biphasic shocks). Neurons were selected for data collection if they were activated antidromically or if they were located in close proximity (<0.5-mm) to an antidromically-activated neuron. Standard tests for antidromic identification were used: constant antidromic latency (<0.2-ms jitter), reliable following of a high-frequency train of stimuli (three or four shocks at 200-Hz), and collision of antidromic spikes with spontaneously occurring spikes (Fuller and Schlag, 1976; Turner and Delong, 2000).

Neuronal activity was collected while the animal performed the step-tracking task. The microelectrode signal was amplified ×10^4^ and bandpass filtered (0.3–10 KHz, DAM-80, WPI Inc.). The action potentials of single neurons (sampled at 60-kHz) were discriminated on-line using template-based spike sorting (MultiSpike Detector, Alpha Omega Engineering, Nazareth, Israel). The timing of detected spikes and of relevant task events was sampled digitally at 1 kHz and saved to disk for offline analysis. EMG signals were differentially amplified (gain = 10-K), band-pass filtered (20-Hz to 5-kHz), rectified and then low-pass filtered (100-Hz). EMG data were collected during only a subset of data recording sessions. (No usable EMG signal was available in monkey L after MPTP administration.) Analog data reflecting angular position of the manipulandum (i.e., joint angle), the torque produced by the motor, and EMG were digitized at either 200-Hz (monkey L) or 500-Hz (monkey V).

### Administration of MPTP

After an adequate number of neurons were sampled from the neurologically-normal state, a hemiparkinsonian syndrome was induced by injection of MPTP into the left internal carotid artery [0.5-mg/kg, (Bankiewicz et al., 1986; Wu et al., 2007)]. This model of parkinsonism was chosen to facilitate care of the animals during the months-long period of post-intoxication recording and to increase the likelihood that animals would continue performing the operant task following intoxication (Bankiewicz et al., 2001). The MPTP administration procedure was performed under general anesthesia (1–3% Isoflurane) and prophylactic antibiotics and analgesics were administered post-surgically. Both animals developed stable signs of parkinsonism contralateral to the infusion (i.e., on the right side of the body). Quantitative measures of the severity of parkinsonism, of its impact on task performance, and histologic evidence of dopamine depletion are documented in a previous report (Pasquereau and Turner, 2011). Post-MPTP recording sessions started >30-days after MPTP administration.

### Histology

After the last recording session, each monkey was given a lethal dose of sodium pentobarbital and was perfused transcardially with saline followed by 10% formalin in phosphate buffer and then sucrose. The brains were processed histologically to localize microelectrode tracks (using cresyl violet staining) and to document the loss of dopaminergic cells in the substantia nigra *pars compacta* (SNc) using tyrosine hydroxylase (TH) immunochemistry (See Pasquereau and Turner (2011) for details concerning histologic results).

### Data analysis

The present report addresses only the short-latency joint movements and changes in neuronal activity evoked by torque perturbation during the start-position hold period. The general features of task performance before and after MPTP have been reported previously (Pasquereau and Turner, 2011).

The digitized signal reflecting manipulandum angle was filtered and differentiated [low-pass 25-Hz (Hamming, 1983)]. The onset of torque-evoked movement, peak velocity, and movement termination were detected automatically using angle, velocity, and duration criteria. A velocity threshold of 5-deg/s was used to detect movement. Session-by-session means of kinematic measures were entered into Three-Way ANOVAs to test for effects of MPTP administration, movement direction and animal.

EMG data were analyzed using methods similar to those described previously (Turner et al., 1995). For each muscle, peri-torque means of the digitized EMG signals were constructed for all valid torque perturbations in each direction (flexion and extension). The signal was normalized by subtracting the background activity recorded 200-ms prior to the onset of the torque perturbation.

Neuronal data were screened based on the location, quality, and duration of the recording. Data were included in the analysis database if they met the following criteria: (1) The recording was obtained from a location within 3-mm of the anterior bank of the central sulcus from which movements of the arm could be evoked by microstimulation (i.e., <40-μA, 10 pulses at 300-Hz). (2) Adequate single unit isolation was maintained throughout the recording. Isolation was controlled during acquisition by adjusting electrode position and spike sorting. Adequate isolation was verified off-line by testing whether a neuron's inter-spike intervals (ISIs) obeyed a refractory period (>2-ms). (3) Neurons were studied if they were either responsive to antidromic stimulation or were encountered within 0.5-mm of an antidromically activated neuron.

The analyses presented here were performed on data extracted from completely different task time periods than those used in our previous publication (Pasquereau and Turner, 2011). In addition, not all neurons were recorded from under all task conditions, so the neuronal populations studied here overlap only partially with those used in (Pasquereau and Turner, 2011).

For each torque perturbation, continuous neuronal activation functions [spike density functions (SDFs)] were generated by convolving a spike train's delta function (1-ms resolution) with an asymmetric Gamma function kernel (*k* = 1.5 and θ = 20). SDFs are traditionally constructed as a sum of Gaussian functions centered on the times of each discriminated action potential (Szucs, 1998). Gaussian functions, however, exert influence backward in time (Thompson et al., 1996; Isoda and Hikosaka, 2008; Heitz et al., 2010) such that SDFs constructed using a Gaussian kernel occasionally gave spurious results, indicating that torque-evoked responses began prior to torque onset. The Gamma function, which approximates the timecourse of a postsynaptic potential, avoided this problem by exerting influence only forward in time. Mean peri-torque SDFs (averaged across trials) were constructed separately for the two torque directions for neurons studied during at least 5 repetitions of each torque direction. A neuron's baseline firing rate was calculated as the mean of the SDFs across the 500-ms epoch immediately preceding torque pulse onset. Phasic responses to a torque perturbation were detected by comparing SDF values (millisecond-by-millisecond) during a post-torque epoch (200-ms) relative to a cell's baseline firing rate (2-tailed *t*-test). The threshold for significance was adjusted to account for multiple comparisons [*p* < 0.01/(200-ms epoch/40-ms gamma filter half-width = 5 independent comparisons) = 0.002]. A neuron was judged to be torque-related if it generated a significant post-torque response for at least one movement direction. Response onset times, defined as the time at which the SDF first crossed the *p* = 0.002 threshold, were determined separately for each torque direction. For comparisons between neuronal populations, a cell's earliest response onset across directions was used as that cell's latency. We only analyzed cortical responses that began at relatively short latencies (between 20–60-ms), so as to exclude responses related to volitional compensatory movements (Evarts, 1973; Pruszynski et al., 2011b). The time of offset of a neuronal response was calculated using a similar method, searching for the time point at which the SDF first returned within the *p* = 0.002 threshold relative to baseline firing rate.

The magnitude of a torque-evoked response was measured using three separate measures. First, the mean firing rate during the response was calculated (i.e., mean of the SDF between times of response onset and offset). Second, we calculated the maximum change of firing rate away from baseline between times of response onset and offset. And third, we calculated the area under the curve in the SDF between times of response onset and offset. The temporal dispersion of a response was measured as its full-width half-maximum (FWHM).

The directional selectivity of torque-evoked responses was parameterized using a directional selectivity index (DSI) (Suarez et al., 1995): DSI = |1 - (NP/*P*)|, where NP equals the maximum change infiring rate from baseline in the non-preferred direction and *P* equals the maximum change in firing rate in the preferred direction during the post-torque period (200-ms).The preferred direction is defined as the direction that elicits the largest change from baseline firing rate (either positive or negative).By this convention, DSI = 0 meant that the response to torque perturbation was equal for both directions and therefore was not directionally selective (classified as *non-directional)*. DSI ≈1 meant that the cell response (either increase or decrease in firing) was present for only one movement direction (classified as *unidirectional activity*). A cell's torque response was considered directionally selective if the DSI was >0.50 (i.e., P/2>NP). Responses with DSIs between 0.5 and 1 were defined as *bidirectional* whereas those with DSIs >1 were considered *reciprocal* (changes of opposite sign for opposing directions of movement).

To study MPTP-related changes for different subsets of cells, we corrected for multiple comparisons using the Bonferroni–Holm method (Holm, 1979). Then, two separate analyses were performed to determine if MPTP-induced alterations in neuronal responses correlated with behavioral measures of rigidity. The first analysis tested for relationships across recording sessions. We tested for correlations between mean measures of neuronal responses (magnitudes and latencies) and mean measures of reflex movements for those sessions (behavioral indexes: peak velocity of movements and magnitude of EMG responses). Spearman's rank correlations were performed and the threshold for significance was adjusted to account for multiple comparisons [*p* < 0.05/(4 comparisons) = 0.0125]. The second analysis tested for relationships within individual recording sessions. We tested for correlations between a single neuron's trial-to-trial response latency and an animal's trial-to-trial response to torque perturbations (peak velocity and EMG). Spearman's rank correlations were performed for individual cells and results were compared between populations recorded pre- and post-MPTP.

## Results

### Database

Single unit recordings were obtained from the arm-related areas of the left M1 of two monkeys. A total of 227 neurons were studied in the neurologically normal state. Of these, 66 were activated antidromically from peduncle stimulation (PTNs: 49 in monkey V and 17 in monkey L; Table 1) and 56 were activated from the putamen (CSNs: 31 in monkey V and 25 in monkey L). Of the 232 neurons collected during the post-MPTP period, 65 were PTNs (54 in monkey V and 11 in monkey L) and 58 were CSNs (48 in monkey V and 10 in monkey L). Only 3 cells were activated antidromically from both the putamen and the peduncle (0.5% of neurons studied). These three cells were included in the general “M1” category (i.e., all cells studied including PTNs, CSNs and non-activated cells), but they were excluded from “PTN” and “CSN” categories. The remainder of the neurons (105 pre-MPTP and 109 post-MPTP) were not activated antidromically (NA), but were recorded either at the same time as PT or CS recordings or were sampled within 0.5-mm of an antidromically activated neuron along the same microelectrode track.

**Table 1:** **Effects of MPTP on two distinct subpopulations of M1 cells**.

		**M1**	**PTN**	**CSN**
Number of cells	Pre-MPTP	227	66	56
	Post-MPTP	232	65	58
Baseline firing rate (Sp/s)	Pre-MPTP	11.8 ± 10.3	18.3 ± 8.6	3.2 ± 4.0
	Post-MPTP	9.5 ± 9.2^*^	13.9 ± 9.6^***^	4.2 ± 4.8
Antidromic latency (ms)	Pre-MPTP	3.3 ± 2.3	1.9 ± 0.9	5.1 ± 2.3
	Post-MPTP	3.6 ± 3.1	1.9 ± 0.8	6.1 ± 3.4^**^
Torque-related cells	Pre-MPTP	141/227 (62%)	43/66 (65%)	19/56 (34%)
	Post-MPTP	143/232 (62%)	46/65 (71%)	16/58 (28%)
Ratio of excitatory responses (Flexion and Extension)	Pre-MPTP	91/111 and 95/106 (86%)	19/34 and 28/33 (70%)	13/13 and 10/11 (96%)
	Post-MPTP	105/119 and 95/108 (88%)	26/35 and 24/32 (75%)	11/13 and 8/9 (86%)^##^

Mean values ± SD before and after MPTP treatment are calculated for all M1 cells (left), for PTNs (middle), and for CSNs (right).

* p < 0.05,

** p < 0.01,

*** *p < 0.001 (Mann–Whitney U-test);* and

## p < 0.01 (χ^2^ test). All statistical results in this table compare results within the indicated category for pre- vs. post-MPTP periods.

Paralleling our previous report (Pasquereau and Turner, 2011), in neurologically-normal animals, resting neuronal firing rates measured immediately prior to torque onset were markedly higher for PTNs (mean rate: 16.3-spikes/s, range: 0.2–45; Table 1) than for CSNs (mean rate: 3.6-spikes/s, range: 0–22; Mann–Whitney *U*-test, *p* < 0.001). This result is consistent with previous descriptions of the marked differences in resting firing rates between intra-telencephalic-like CSNs and PTNs (Bauswein et al., 1989; Turner and Delong, 2000).

Also consistent with previous reports (Pasquereau and Turner, 2011) the spontaneous activity of the general population of M1 cells decreased by 17% after MPTP treatment (Mann–Whitney *U*-test, *p* < 0.05/2). MPTP had markedly different effects on the two identified neuronal populations. The pre-torque firing rate of PTNs was reduced by 27% following MPTP treatment (Mann–Whitney *U*-test, *p* < 0.001; Table 1) whereas the mean activity of CSNs remained unchanged (Mann–Whitney *U*-test, *p* = 0.5). The antidromic latencies of PTNs remained unchanged between pre- and post-MPTP periods (mean = 1.9-ms; Mann–Whitney *U*-test, *p* = 0.9; Table 1) whereas those of CSNs were significantly longer following MPTP administration (means = 5.1 and 6.1-ms, pre- and post-MPTP, respectively; Mann–Whitney *U*-test, *p* < 0.05/3).

### Behavioral effects of MPTP

Intracarotid administration of MPTP rendered the animals moderately parkinsonian as evidenced by increased reaction times, decreased movement velocities and reduced movement extents in the behavioral task [for details, see Pasquereau and Turner (2011)]. Monkeys showed limited variation in the severity of these impairments throughout the post-MPTP recording period (maximum 117-days).

**Figure 1:**
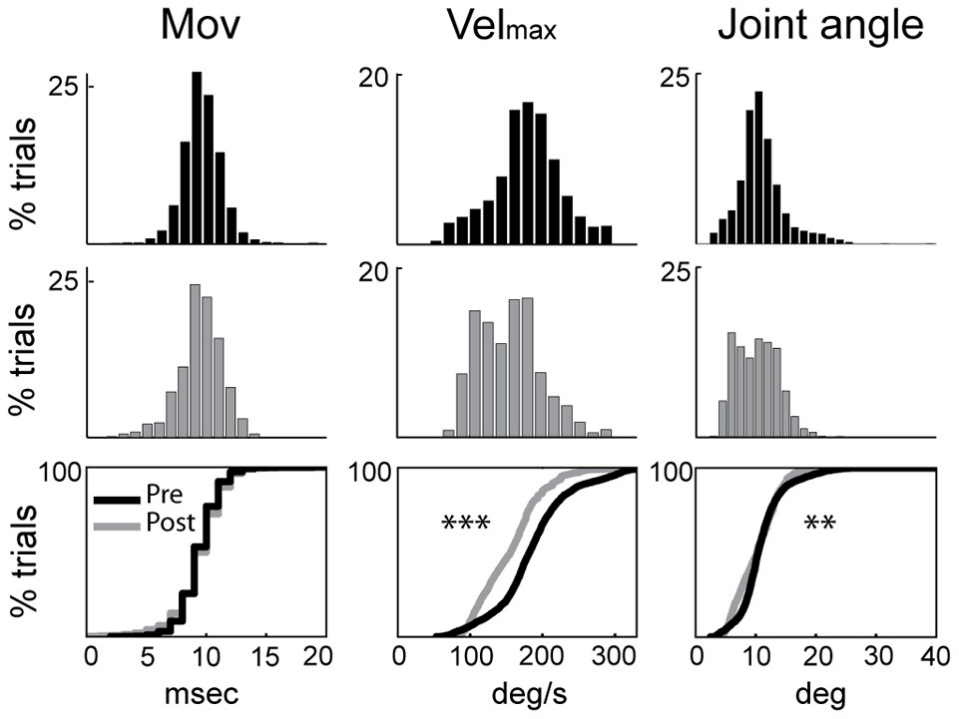
**Rigidity was increased following MPTP administration**. Kinematic measures (cross-session means) from pre-MPTP (black) and post-MPTP (gray) periods for flexion movements following torque perturbations. Cumulative distributions are shown at the bottom. Movement onsets (*Mov*), peak velocities (*Vel*_max_), and movement amplitudes (*Joint angle*) were compared between MPTP states (ANOVA; ^**^p < 0.01, ^***^p < 0.001).

Induction of parkinsonism also led to a slowing of torque-induced displacements of the arm (ANOVA; *F* > 515, *p* < 0.001; Figure 1) and a reduction in movement amplitude (*F* > 49, *p* < 0.01). More specifically, the mean peak velocity and the amplitude of torque-induced displacements averaged across recording sessions were reduced by 16.2% and 6.6%, respectively, following MPTP administration, consistent with an increase of rigidity in the parkinsonian condition. The latencies of torque-induced displacements remained unmodified post-MPTP (*F* < 0.2 and *p* > 0.05; Figure 1), but we found significant differences in the effects of MPTP for the two torque directions (*F* > 94 and *p* < 0.001). Because of this, data were analyzed separately for flexion and extension torque directions.

**Figure 2:**
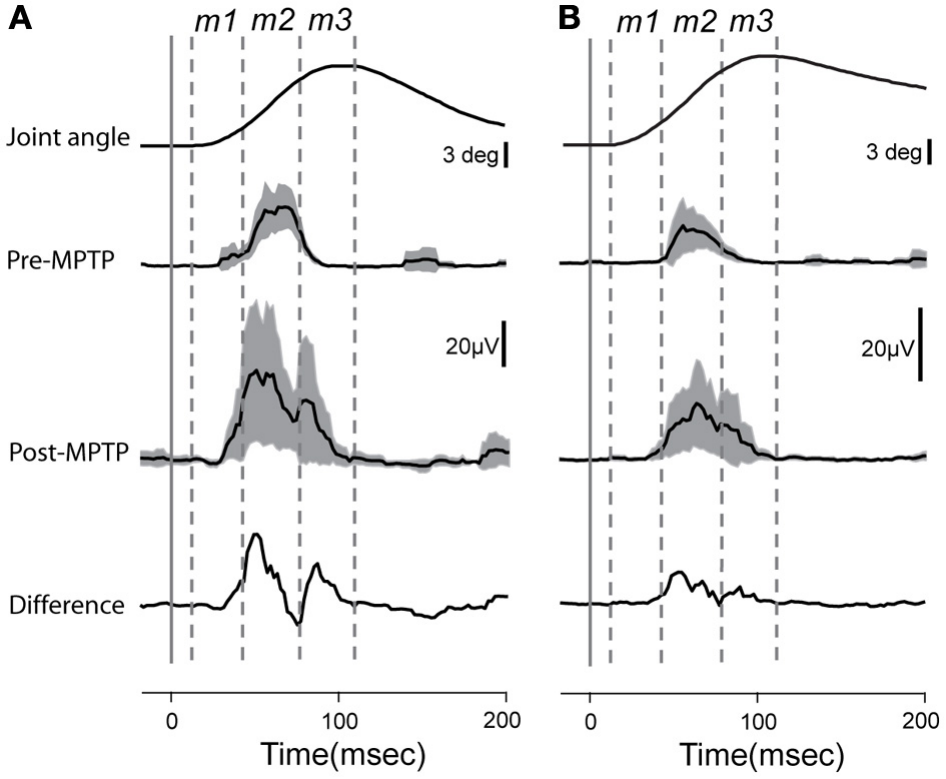
**Averaged and rectified grand-average EMG responses (±95% confidence intervals) to elbow flexion from triceps longus (A) and triceps lateralis (B)**. Lower traces reflect the differences between EMG responses recorded before or after MPTP administration. Time zero indicates the time at which the torque impulse was turned on to initiate the arm displacement. Vertical lines show the putative onset of each muscle component (*m1*, *m2*, and *m3*).

EMG responses to sudden, unpredictable muscle stretches were analyzed in their separate latency components (Figure 2). The short-latency response defined as the *m1* component (muscle activity occurring between 15 ± 3 and 40 ± 4-ms post-torque) was poorly formed and generally much smaller than the long-latency response (*m2*; 41 ± 4 and 80 ± 7-ms post-torque). Following the administration of MPTP, the *m2* component was markedly larger (Kolmogorov two-sample test, *p* < 0.05; Figure 2). The total duration of EMG responses to torque perturbations also increased (from 57 ± 6 to 79 ± 3-ms; Kolmogorov two-sample test, *p* < 0.001), primarily due to the appearance of an *m3* component. The *m1* short-latency response remained weak in all the case, and latencies of the earliest EMG responses showed no significant change (*p* > 0.05).

### Torque-evoked neuronal responses – prevalence

**Figure 3:**
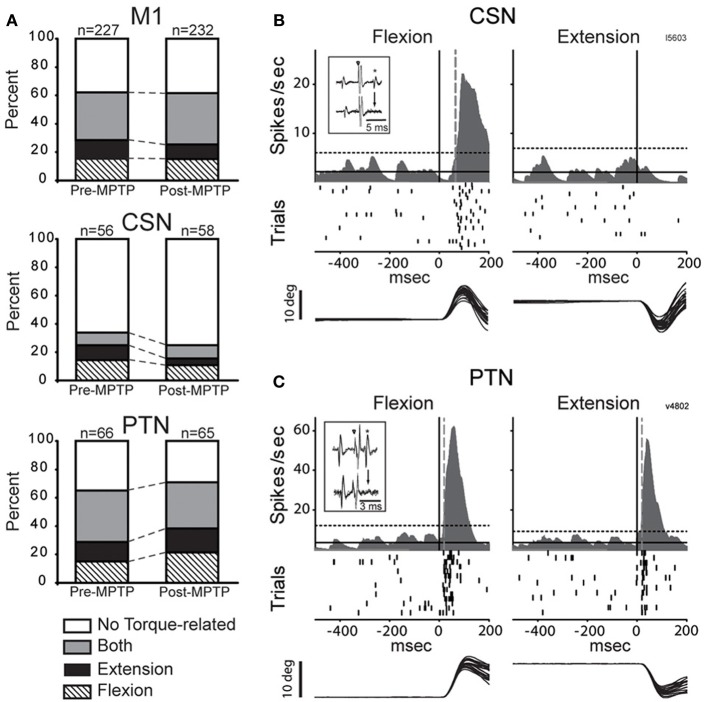
**MPTP did not alter torque responsiveness. (A)** The fractions of neurons that were torque-related to one or both stretch directions (flexion and extension) did not change following MPTP administration for the general population of M1 cells (top), for CSNs (middle), or for PTNs (bottom) (χ^2^ test, *p* > 0.05/3). **(B,C)** Representative short latency responses to torque perturbations for CSNs **(B)** and PTNs **(C)**. The torque responses of CSNs tended to be small in magnitude and in one direction only, whereas the responses of PTNs were most commonly sensitive to both directions. Mean SDFs, rasters, and overlaid traces of single trial joint are aligned on the onset of flexing or extending torques (*vertical black lines*). The *vertical gray dotted lines* show neuronal response latencies detected using significant threshold computed from the pre-torque period (*horizontal dotted lines*, *p* < 0.01/(5 comparisons) = 0.002). *Inset figures* in each panel illustrate antidromic activation and collision tests for the neuron that has its torque response shown.

A large fraction of the general population of M1 cells (62%, 284/459, of all CSNs, PTNs, and NA cells) responded to torque perturbations with a phasic response at short latency (<60-ms, Figure 3A). Half of these torque-related cells (124/284, 44%) responded for only one direction of rotation (flexion or extension). Figures 3B,C illustrate examples of neuronal responses to torques in which a CSN responded to flexion torques only (3B), and a PTN that responded to both flexion and extension torques (3C). Monophasic increases in firing constituted a large fraction of the torque response in M1 (87%) whereas torque-evoked decreases in firing were observed infrequently (13% of all M1 neurons; Table 1). Consistent with previous observations (Turner and Delong, 2000), PTNs were more responsive to proprioceptive stimulation than CSNs. Torque responses were observed in 68% (89/131) of PTNs but only 31% (35/114) of CSNs (χ^2^ = 32.3, *p* < 0.001; Table 1 and Figure 3A).

### Torque-evoked neuronal responses – pre- vs. post-MPTP

Contrary to predictions from a simple model for exaggerated LLSRs in PD (see Introduction), induction of parkinsonism did not increase the prevalence of torque responses in the general population of M1 cells (62% for both states), in CSNs (26%), or in PTNs (68%; χ^2^ < 6.4 *p* > 0.05/3; Figure 3A, Table 1). These results are broken down in a more fine-grained manner for the two monkeys in the Supplementary Table. The small number of proprioceptive-responsive CSNs studied post-MPTP prevented more in-depth analysis of response timing in this neuronal type.

**Figure 4:**
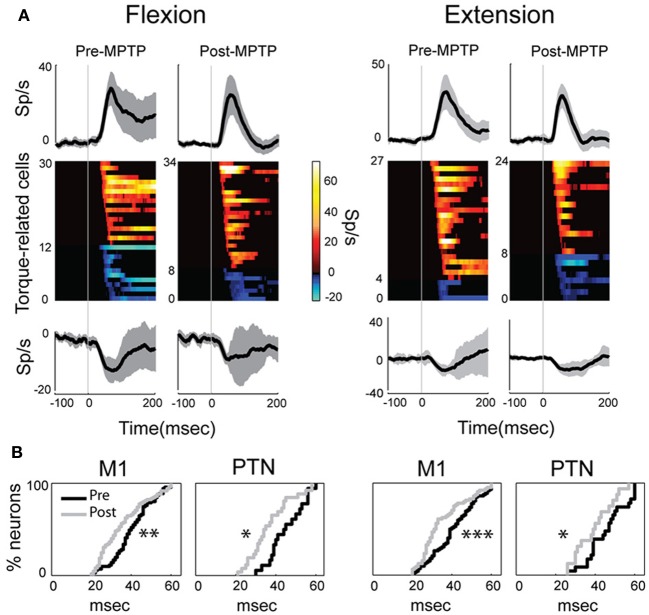
**MPTP altered the M1 response latencies to torque perturbations**. The latency of response shifted significantly in PTNs or in the general population of M1 cells, although its magnitude remained unchanged between MPTP periods. **(A)** Population mean spike density functions (SDFs) averaged across all positive (top) or negative (bottom) phasic responses in PTNs for flexion or extension movements (left and right panels, respectively). Gray shadings and vertical gray lines indicate ± *SE* and the time of torque onset (time 0), respectively. Color plot of all SDFs (one row per PTN) classified neurons according to latency and polarity of responses. Colors along each horizontal band indicate the significant changes in firing rate of one PTN induced by torque perturbation (red-yellow = increases; blue-cyan = decreases; firing rate scale in the bar). Black = no significant change in a SDF. **(B)** Following MPTP, the cumulative distributions for M1 (left) or for PTNs (right) showed a reduction of response latency to torque perturbation (Kolmogorov two-sample test; ^***^p < 0.001, ^**^p < 0.01, ^*^p < 0.05).

Induction of parkinsonism also did not alter the balance of torque-elicited increases and decreases in firing. For PTNs, most torque-evoked changes in activity consisted of an increase in firing (72% of responses; 97 of 134 responses counting responses to flexions and extensions separately; Figure 4A and Table 1). That high prevalence of torque-evoked increases in firing did not differ between pre- and post-MPTP periods (χ^2^ = 0.1, *p* = 0.7; Table 1). The magnitudes of torque-evoked responses also did not differ between pre- and post-MPTP periods. Response magnitudes were compared for M1 cells, and again for PTNs separately, using three different measures of magnitude: (1) the mean change of firing rate relative to pre-torque baseline, (2) the maximum change of firing rate, and (3) the area under the curve in the SDF. None of these comparisons yielded a significant difference between pre- and post-MPTP periods (Kolmogorov two-sample test and Mann–Whitney *U*-test, all *p*'s > 0.15; Figure 4A). To control for the possibility that these results were biased by the MPTP-induced global slowing of torque-induced displacements of the arm (see *Behavioral effects of MPTP*, above), we selected subsets of recording sessions from pre- and post-MPTP sessions in which velocities were equivalent (range: 130–200 deg/s, Mann–Whitney *U*-test, *p* > 0.05; Supplementary Figure 1). We confirmed our main result in this subset by observing that MPTP did not change the magnitude of torque-evoked responses in M1 (Mann–Whitney *U*-test, all *p*'s > 0.19).

The latencies of torque-evoked responses, however, were significantly shorter following MPTP administration. This observation held true for the general population of M1 neurons and specifically for PTNs, and for responses to flexions and extensions, all considered separately (Kolmogorov two-sample test, all *p*'s < 0.05; Figure 4B). For PTNs, the mean latency of responses was reduced by 17% from a pre-MPTP value of 46-ms (45-ms for flexions, and 46-ms for extensions) to a post-MPTP value of 38-ms (37-ms for flexions, and 39-ms for extensions). Similarly, for the general population of M1 neurons, mean response latencies were reduced by 15% from 41-ms pre-MPTP (40-ms for flexions, and 42-ms for extensions) to 35-ms post-MPTP (36-ms for flexions, and 35-ms for extensions; Kolmogorov two-sample test, *p* < 0.01). M1 response latencies were reduced by similar degrees for flexion and extension directions (Kolmogorov two-sample test, *p* = 0.52; Figure 4B). This latency shortening effect could not be attributed to history effects (e.g., increasing experience by the animal or cumulative recording tracks) because we found no correlation between the latencies of torque-evoked responses and the days of training an animal experienced (monkey V: Spearman |rho|= 0.02 *p* = 0.85; monkey L: Spearman |rho|= 0.08 *p* = 0.68).

In addition to the shift in latencies, torque-evoked increases in PTN activity were more tightly synchronized or time-locked to the torque perturbation following MPTP (Figure 4A). For these responses, the response FWHM was reduced by 31% from a mean of 73-ms (80-ms for flexion, and 66-ms for extension) pre-MPTP to 50-ms (54-ms for flexion, and 46-ms for extension) post-MPTP (Mann–Whitney *U*-test, *p*'s < 0.05).

### Response directionality – pre- vs. post-MPTP

**Figure 5:**
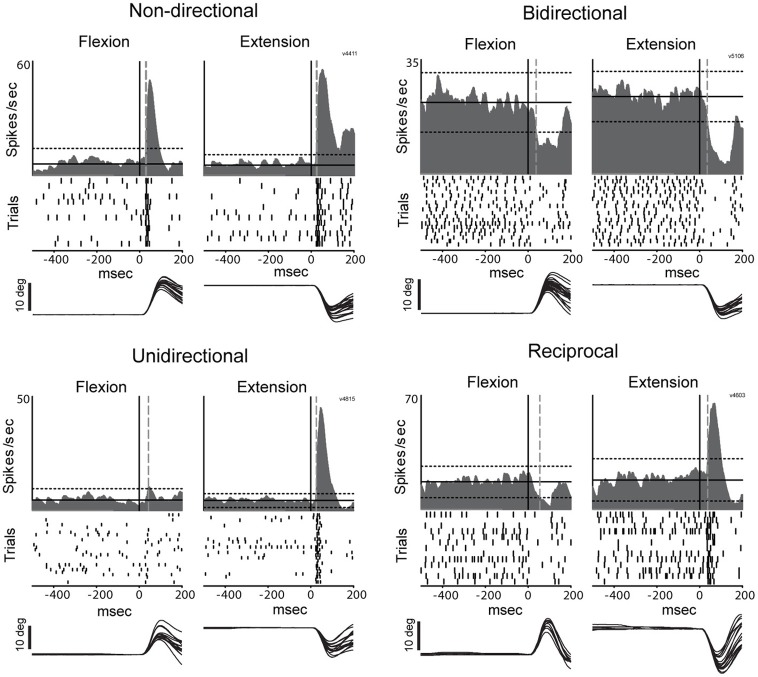
**Representative examples of directional peri-torque activity in PTNs**. *Non-directional* neuron similarly responded to torque perturbation for both directions (flexion and extension; DSI = 0). *Unidirectional* activity indicates that torque response only occurred for the preferred direction (the direction associated with a maximal change in firing; DSI = 1). *Bidirectional* neuron was considered directionally selective with a change in firing for the preferred direction ≥2 times the magnitude of that for the opposing direction (0.5 < DSI < 1). *Reciprocal* responses indicate that changes in firing were opposite sign (increase and decrease in firing for preferred and non-preferred directions,respectively) for opposing directions of movement (DSI >1). These figures follow the conventions of Figure 3 to illustrate mean SDFs, rasters, and arm positions.

**Figure 6:**
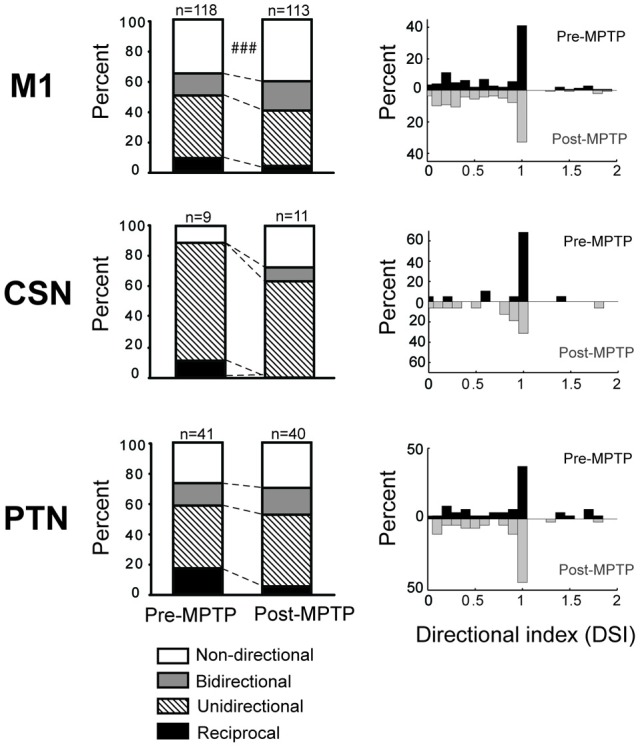
**MPTP altered the directionally selective responses**. Compared to the pre-MPTP period, the short latency torque responses of M1 were directionally selective less frequently following the MPTP treatment: a smaller proportion of neurons had reciprocal or unidirectional activities, whereas the responses were most commonly non-directional or bidirectional. These modifications were partly attributable to PTNs, for instance, whose reciprocal responses were less frequent in post-MPTP. The small number of CSNs showing directionally selective responses further limited the statistical analysis. χ^2^ test; ^###^*p* < 0.001.

In the general population of M1 cells, the directional selectivity of torque responses was reduced following MPTP. Prior to MPTP, a large proportion of the torque-responsive cells in M1 responded differently for the two directions of torque perturbation (Figure 5; 143/231, 62% of all torque-responsive cells). By classifying the torque-evoked responses of M1 neurons according to their directional selectivity indices (DSI, *Methods*), it became clear that highly directional (reciprocal and unidirectional) response types became less common following MPTP administration (10% reduction in incidence), and bi-directional or non-directional response types became more common (10% increase in incidence; χ^2^ = 22.2, *p* < 0.001; ^###^ in Figure 6). Non-significant reductions (*p* > 0.05/2, likely due to small N) in the incidence of highly directional response patterns were observed in CSN and PTN sub-populations. This general decrease in directional selectivity could not be attributed directly to MPTP-induced changes in resting firing rates because pre-torque mean firing rates did not differ between the different directional categories (One-Way ANOVA, *F* = 0.77 and *p* = 0.46). In addition, EMG responses to torque perturbations did not show alterations in directional selectivity or sign of co-contraction between antagonist muscles (Supplementary Figure 2).

### Analysis of neural-behavioral correlations

Although torque-evoked cortical responses, movement velocity, and the *m2* component of EMG reflex activity were all affected by MPTP administration, we found no correlation between the modifications in M1 responses and variations in the LLSR. That question was addressed first by testing for correlations between session-by-session measures of the mean PTN response latency and mean measures of the LLSR (i.e., peak velocity of movements, and magnitude of the *m2* muscle component). This analysis was restricted to the relatively homogenous population of PTNs because their activity was known to be affected by MPTP administration. No significant correlation was found for either analysis (Vel_max_, Spearman |rho| < 0.18 *p* > 0.05; or *m2*_max_, |rho| < 0.26 *p* > 0.05). A second approach searched for correlations trial-by-trial between torque-evoked neuronal responses and measures of the LLSR (Vel_max_ or *m2*_max_). Measures of PTN response latencies did not correlate with any of the measures of LLSR (Spearman |rho| < 0.34; *p* > 0.05). Similar negative results were obtained from correlation analyses of the general population of M1 cells (results not shown).

## Discussion

The present study addresses a long-standing but untested hypothesis for why the long-latency component of the stretch reflex is exaggerated in Parkinson's disease. Despite the fact that exaggeration of the LLSR is a significant contributor to parkinsonian rigidity (Lee and Tatton, 1975; Mortimer and Webster, 1979; Tatton et al., 1984; Xia et al., 2009), the CNS correlates of the parkinsonian stretch reflex have seldom been examined (Rossini et al., 1989; Aminoff et al., 1997) and never, to our knowledge, using single unit recording. One straightforward hypothesis states that the LLSR is exaggerated in PD because of increased corticospinal sensitivity to muscle stretch (Rothwell et al., 1983). Alternative hypotheses implicate increased gain in slow-conducting spinal reflex pathways (Berardelli et al., 1983). We tested the trans-cortical hypothesis by measuring the stretch-evoked responses of identified sub-populations of M1 neurons prior to and following the induction of parkinsonism. Contrary to the predictions of the trans-cortical model, we did not observe an increase in M1 responsiveness to proprioceptive stimuli. Rather, the MPTP-induced increase in rigidity and LLSR was associated with shortened M1 response latency and a reduction in directional selectivity. The sections below address each of these findings in turn.

### Absence of increased response prevalence or magnitude

We found no evidence that the induction of parkinsonism lead to a greater incidence of neuronal responses to proprioceptive stimuli in the population of M1 neurons, an increase in the balance of torque-evoked increases over decreases, or an increase in the magnitude of torque-evoked responses in individual M1 neurons. These observations run contrary to predictions of the simple trans-cortical model for the exaggerated LLSR of PD. In their key study of the LLSR in PD patients, Rothwell et al. stated that “if the stretch reflex excitability is indeed enhanced in PD, the gain must be increased at some central site”(Rothwell et al., 1983, p. 35). The M1, and its population of PTNs in particular, is one of the few central nodes in the circuit that mediates the LLSR. A straightforward interpretation of Rothwell et al.'s statement predicts that neurons in this circuit will show an increased prevalence of stretch-evoked neuronal responses or increased neuronal response magnitudes. We found no evidence of either in the activity of single units in M1.

Although inconsistent with the simple trans-cortical hypothesis, this result agrees with the abundant evidence that somatosensory-evoked responses are not enhanced in the sensorimotor cortex of parkinsonian subjects (Rossini et al., 1989; Aminoff et al., 1997; Rickards and Cody, 1997; Boecker et al., 1999; Lewis and Byblow, 2002; Schrader et al., 2008). The classical rate model of PD pathophysiology also proposes that excessive inhibitory outflow from the parkinsonian BG attenuates thalamo-cortical responses (Delong and Wichmann, 2007). Despite the limitations of the rate model (Montgomery, 2007), evidence continues to support the model's prediction that parkinsonism is associated with a reduction in M1 excitability (Lefaucheur, 2005; Brown et al., 2009; Pasquereau and Turner, 2011; Viaro et al., 2011; Leon-Sarmiento et al., 2013). From this perspective, it might be seen as surprising that we did not observe more profound *decreases* in the prevalence or magnitude of M1 responses to proprioceptive stimulation. Our failure to observe an attenuation of proprioceptive responsiveness, as predicted by indirect measures of brain activity (Rossini et al., 1989; Aminoff et al., 1997; Rickards and Cody, 1997; Boecker et al., 1999; Lewis and Byblow, 2002; Schrader et al., 2008), may be explained by the idea that M1 is organized into dissociable sub-circuits that are affected differently with the induction of parkinsonism (Shepherd, 2013).

Numerous studies have reported that neurons in BG structures and in the motor thalamus of parkinsonian animals show marked increases in the prevalence and magnitude of proprioceptive responses (Filion et al., 1988; Bergman et al., 1994; Pessiglione et al., 2005; Bronfeld and Bar-Gad, 2011). Our results suggest that this exaggerated proprioceptive responsiveness in subcortical structures is not relayed to cortex in any simple way. This result is congruent with other lines of evidence that indicate that the BG-thalamo-cortical pathway does not operate as a simple driver of cortical activity (Inase et al., 1996; Rubin and Terman, 2004; Kuramoto et al., 2009; Goldberg and Fee, 2012).

Our observation of an elevated LLSR (Figure 2) combined with no change in prevalence or magnitude of stretch-evoked neuronal responses in PTNs is consistent with the view that the alteration in reflex function that mediates the exaggerated LLSR of PD is localized to the spinal cord (Simonetta Moreau et al., 2002; Marchand-Pauvert et al., 2011; Raoul et al., 2012). Several abnormalities in segmental function have been observed in PD and there is no consensus as to which of them contribute most significantly to the LLSR or rigidity. Some studies implicate slow-conducting group II spindle afferents (Cody et al., 1986; Marchand-Pauvert et al., 2011). Others suggest impairments in inhibition (Tsai et al., 1997; Meunier et al., 2000), perhaps via underactivation of the IB interneuron (Delwaide et al., 1991), or, alternatively, an abnormality in homosynaptic depression (Raoul et al., 2012). For all of these, dysfunction within the BG may affect segmental motor function via descending BG projections to the pedunculopontine nucleus and from there to spinal cord projecting brainstem nuclei (Delwaide et al., 2000). Degeneration of the noradrenergic projection to the spinal cord may also play an important role (Simonetta Moreau et al., 2002).

### Altered response latencies and directionality

The stretch-evoked neuronal responses in M1 began at shorter latencies following MPTP administration (Figure 4B). The fact that very similar latency shifts were observed for responses to torque perturbations in the flexion and extension directions suggests that the latency shift was a primary effect of parkinsonism and not a by-product of altered postural bias or background muscle activity. The mechanisms that might mediate such a shift in response latency remain unclear as do its functional implications.

We also found that the directional specificity of neuronal responses was reduced following MPTP administration (Figure 6). Similar reductions in somatosensory specificity have been reported for neuronal activity in BG structures and in the thalamus of parkinsonian subjects (Filion et al., 1988; Bergman et al., 1994; Pessiglione et al., 2005). Our observation of reduced encoding of movement direction in M1 is consistent with the general concept that a reduction in functional specificity may be an important component of the pathophysiology of PD (Bronfeld and Bar-Gad, 2011). More specifically, the increased prevalence of non-directional and bidirectional responses may contribute to the genesis of EMG “shortening reactions,” which are abnormal muscle reflex responses to passive joint rotation that appear in the muscles that are shortened by the rotation (Andrews et al., 1972; Berardelli and Hallett, 1984). Recent evidence implicates the shortening reaction in the lead-pipe nature of parkinsonian rigidity (Xia et al., 2009, 2011). The deficient directional specificity observed in M1 may contribute to the genesis of shortening reactions by producing corticospinal excitation of segmental circuit elements that should, under normal conditions, be suppressed (e.g., in the motor neuron pool of the shortened muscle). A similar mechanism may account for the more transient responses observed following MPTP administration (Figure 4).

### Methodologic considerations

This is the first study to our knowledge to examine single unit activity in M1 related to the exaggerated LLSR of parkinsonism. We examined the torque-evoked responses of M1 neurons while our research subjects were engaged in a behavioral task that required postural stabilization. This approach allowed us to maintain relative control over the behavioral state of our animal subjects across the induction of moderate parkinsonism. We used the well-established non-human primate MPTP model of PD (Bankiewicz et al., 1986, 2001) and we studied the activity of two distinct sub-populations of cortical neurons, PTNs and CSNs, as identified by antidromic activation. The divergent effects of MPTP intoxication on the two neuronal populations is consistent with the view that the two play different roles in the pathophysiology of PD (Shepherd, 2013).

It is important to recognize several limitation to the methodology used. First, we did not pre-load the muscle to be stretched or control for the level of muscle activity prior to delivery of torque perturbations, as is often done in studies of the stretch reflex (Rothwell et al., 1983). It is unlikely that this represents a serious confound for the principal results, however, because the trans-cortical component of the LLSR appears to be insensitive to the pre-perturbation level of muscle activity (Pruszynski et al., 2011b).

Second, the contribution of the trans-cortical pathway to the LLSR appears to vary between effectors, being maximal for finger muscles (Marsden et al., 1983; Noth et al., 1991) and of less importance for muscles around more proximal joints such as the wrist and elbow (Berardelli et al., 1983; Cody et al., 1986). We might have obtained different results if we had studied, for example, the effects in M1 of proprioceptive perturbations delivered to intrinsic muscles of the hand.

Third, the peak velocity and amplitude of torque-evoked joint movements were significantly smaller following MPTP administration (Figure 1). The magnitude of the LLSR is known to be modulated in proportion to the velocity of muscle stretches (Rothwell et al., 1983; Powell et al., 2012). The stretch-evoked neuronal responses in M1 might have been more numerous or larger in magnitude if the perturbation kinematics had been identical pre- and post-MPTP. However, the difference in kinematics is unlikely to invalidate the significance of our main results. Even when data were selected from subsets of recording sessions that had equivalent velocities, we found that response magnitudes were very similar in the pre-and post-MPTP periods (Supplementary Figure 1).

Fourth, different results might have been observed if we had induced more severe parkinsonian symptoms or bilateral parkinsonism. Although our animals showed clear behavioral and histologic signs of moderate parkinsonism (Pasquereau and Turner, 2011), the symptoms of our animals were not as severe as those rendered by other MPTP intoxication protocols (Bankiewicz et al., 2001; Emborg, 2007).

## Conclusions

In summary, our results are not consistent with the idea that the exaggerated LLSR of PD is mediated by an increase in trans-cortical reflex gain. The most likely alternative is that reflex gain is abnormally increased in slow-conducting segmental pathways, such as those driven by group II spindle afferents (Cody et al., 1986; Marchand-Pauvert et al., 2011). The reduced directional specificity of M1 responses to muscle stretch provides additional evidence for the general breakdown in functional specificity in parkinsonism. This breakdown in directional specificity may contribute to the abnormal shortening reactions that contribute to parkinsonian rigidity.

## Funding

This work was supported by National Institute of Neurological Disorders and Stroke at the National Institutes of Health (grant numbers NS044551 and NS055197 to Robert S. Turner and the Center for Neuroscience Research in Non-human Primates, 1P30NS076405).

### Conflict of interest statement

The authors declare that the research was conducted in the absence of any commercial or financial relationships that could be construed as a potential conflict of interest.
