# Supplementary material for: Primary motor cortex of the parkinsonian monkey: altered neuronal responses to muscle stretch
Source: Front Syst Neurosci. 2013 Nov 26;7:98. doi: 10.3389/fnsys.2013.00098 (PMC3840326; doi:10.3389/fnsys.2013.00098)
Supplement: Supplementary file 1 [file DataSheet1.PDF]

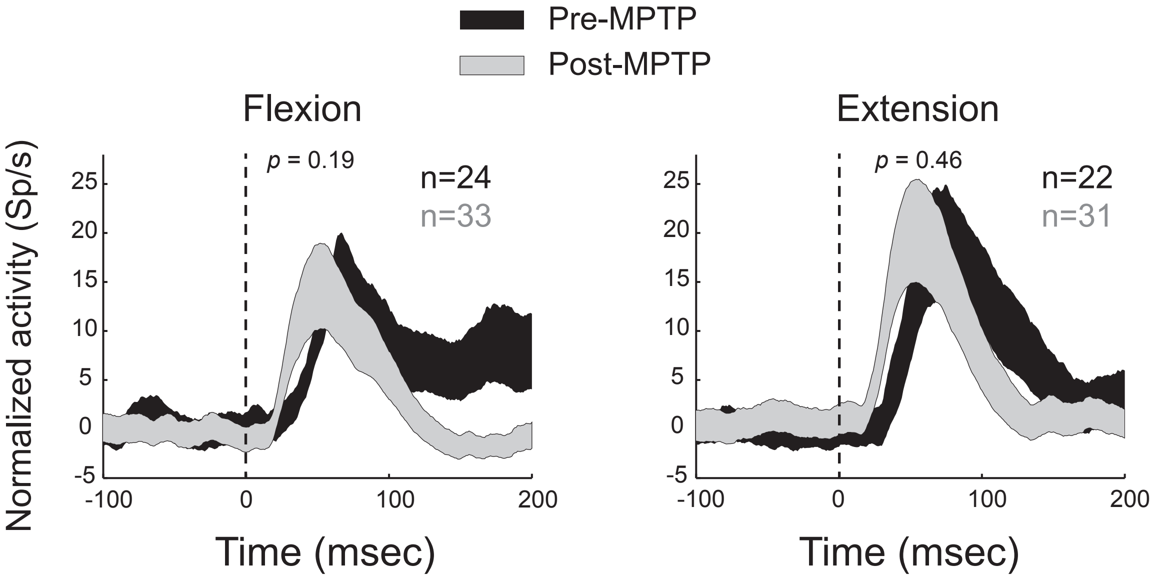

Supplementary figure 1. MPTP did not modify the magnitude of torque-evoked responses. To control for a putative bias relative to the MPTP-induced difference in movement velocity, we compared population-averaged responses (mean ± sem) between MPTP periods only with neurons that were recorded with equivalent torque-induced displacements (range: 130-200 deg/s). For both movement directions, none of these comparisons yielded a significant difference between pre- and post-MPTP periods (Mann-Whitney U-test).

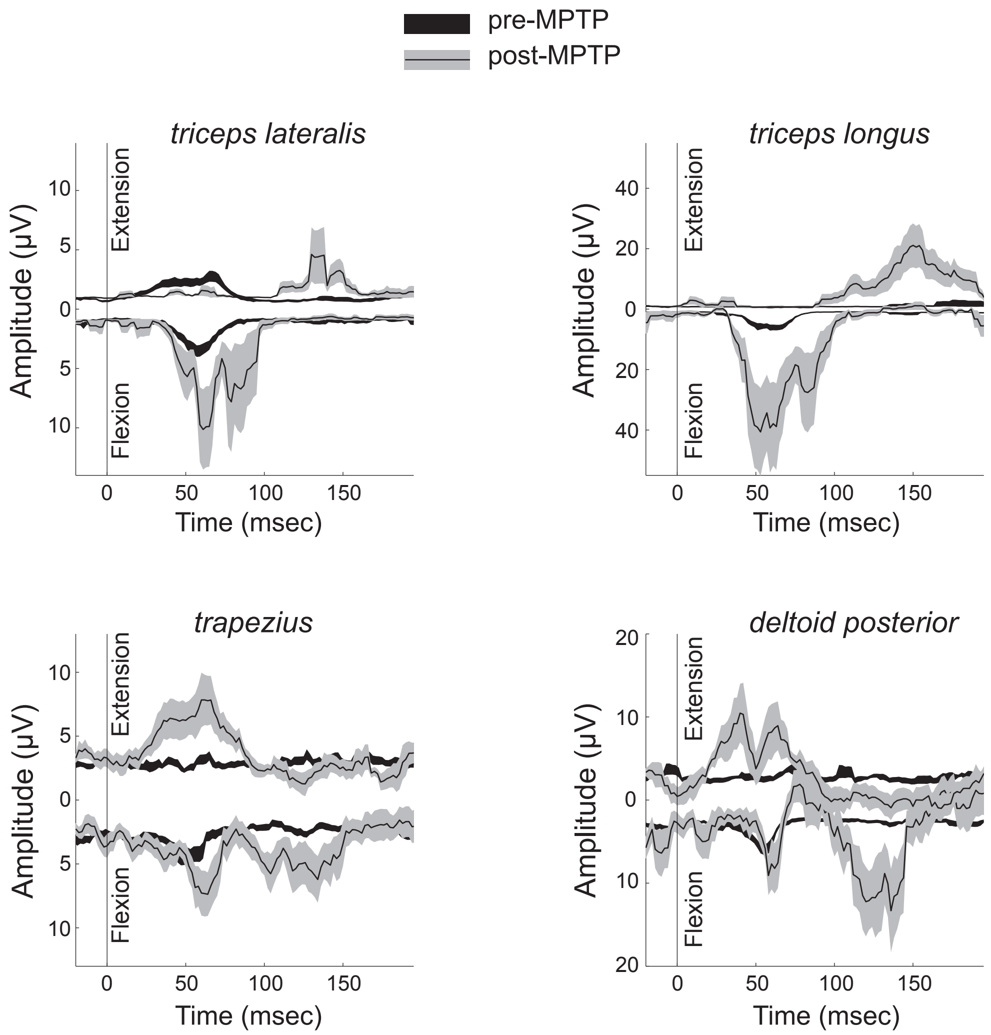

Supplementary figure 2. Averaged and rectified grand-average EMG responses (±95% confidence intervals) to elbow flexion (lower traces) or extension (upper traces) recorded in monkey V. For each muscle, EMG responses show data for the pre- (*black*) and post-MPTP (*grey*) periods. Time zero indicates the time at which the torque impulse was turned on to initiate the arm displacement. No loss of directional selectivity or co-contractions are observed in the present muscle activities.

Supplementary table. Effects of MPTP on neuronal parameters (mean values ± SD) for two distinct monkeys

		Monkey V		Monkey L	
Category of cells	Parameters	pre-MPTP	post-MPTP	pre-MPTP	post-MPTP
M1	Number of cells	112	159	115	73
	Baseline firing rate (Sp/s)	12.6 ± 10.5	8.9 ± 8.4	11.1 ± 10.3	10.22 ± 8.6
	Torque-related cells	84/112 (75%)	108/159 (68%)	57/115 (50%)	35/73 (48%)
	Directional index (DSI)	0.72 ± 0.4	0.66 ± 0.4	0.8 ± 0.5	0.71 ± 0.4
	Flexion:				
	Latency of responses (msec)	39.2 ± 11.3	34.56 ± 12.8	42.4 ± 27.9	37.4 ± 27.1
	Magnitude of responses (Sp/s)	42.4 ± 40.3	37.9 ± 35.6	29.5 ± 21	34.19 ± 32.1
	Response FWHM (msec)	85 ± 33	67 ± 29	101 ± 18	83 ± 31
	Extension:				
	Latency of responses (msec)	40.4 ± 11.7	33.4 ± 15.5	44.3 ± 30	37.5 ± 27.5
	Magnitude of responses (Sp/s)	50.9 ± 40.1	42.6 ± 36	28.4 ± 21.9	29.5 ± 27.4
	Response FWHM (msec)	71 ± 33	67 ± 26	69 ± 16	58 ± 28
PTN	Number of cells	49	54	17	11
	Baseline firing rate (Sp/s)	20.3 ± 8.6	14.8 ± 10	16.2 ± 5.6	12.96 ± 7.8
	Torque-related cells	27/49 (55%)	37/54 (68%)	16/17 (94%)	9/11 (82%)
	Directional index (DSI)	0.73 ± 0.3	0.75 ± 0.4	1.09 ± 0.5	0.71 ± 0.4
	Flexion:				
	Latency of responses (msec)	43.9 ± 8.1	35.5 ± 11.3	48.1 ± 35.7	39.1 ± 18.4
	Magnitude of responses (Sp/s)	33.4 ± 19.4	26.1 ± 15.4	19.4 ± 9.4	26.9 ± 26.5
	Response FWHM (msec)	97 ± 20.3	52 ± 14.2	65 ± 11.9	57 ± 29.3
	Extension:				
	Latency of responses (msec)	44.4 ± 7.5	36.7 ± 15.1	49.1 ± 26.6	42.9 ± 31.2
	Magnitude of responses (Sp/s)	38.8 ± 24.7	29.3 ± 18.6	27.7 ± 13	27.9 ± 23.3
	Response FWHM (msec)	57 ± 21.1	41 ± 13.2	77 ± 15.3	52± 26.2
CSN	Number of cells	31	48	25	10
	Baseline firing rate (Sp/s)	3.5 ± 4.1	3.4 ± 3.5	2.8 ± 3.9	5.1 ± 4.9
	Torque-related cells	14/31 (45%)	10/48 (21%)	5/25 (20%)	6/10 (60%)
	Directional index (DSI)	0.81 ± 0.4	0.83 ± 0.4	0.92 ± 0.3	0.78 ± 0.61
